# Acute Heart Failure Registry: Risk Assessment Model in Decompensated Heart Failure

**DOI:** 10.5935/abc.20160178

**Published:** 2016-12

**Authors:** Anne Delgado, Bruno Rodrigues, Sara Nunes, Rui Baptista, Bruno Marmelo, Davide Moreira, Pedro Gama, Luís Nunes, Oliveira Santos, Costa Cabral

**Affiliations:** 1 Serviço de Cardiologia, Centro Hospitalar Tondela Viseu; Viseu - Portugal; 2 Instituto Politécnico de Castelo Branco - Escola Superior de Gestão; Viseu - Portugal; 3 CNC.IBILI Research Consortium - Faculdade de Medicina Universidade de Coimbra, Viseu - Portugal

**Keywords:** Heart Failure/complications, Prognosis, Acute Coronary Syndrome, Biomarkers, Echocardiography,Doppler

## Abstract

**Background:**

Heart failure (HF) is a highly prevalent syndrome. Although the long-term prognostic factors have been identified in chronic HF, this information is scarcer with respect to patients with acute HF. despite available data in the literature on long-term prognostic factors in chronic HF, data on acute HF patients are more scarce.

**Objectives:**

To develop a predictor of unfavorable prognostic events in patients hospitalized for acute HF syndromes, and to characterize a group at higher risk regarding their clinical characteristics, treatment and outcomes.

**Methods:**

cohort study of 600 patients admitted for acute HF, defined according to the European Society of Cardiology criteria. Primary endpoint for score derivation was defined as all-cause mortality and / or rehospitalization for HF at 12 months. For score validation, the following endpoints were used: all-cause mortality and / or readmission for HF at 6, 12 and 24 months. The exclusion criteria were: high output HF; patients with acute myocardial infraction, acute myocarditis, infectious endocarditis, pulmonary infection, pulmonary artery hypertension and severe mitral stenosis.

**Results:**

505 patients were included, and prognostic predicting factors at 12 months were identified. One or two points were assigned according to the odds ratio (OR) obtained (p < 0.05). After the total score value was determined, a 4-point cut-off was determined for each ROC curve at 12 months. Two groups were formed according to the number of points, group A < 4 points, and group B = 4 points. Group B was composed of older patients, with higher number of comorbidities and predictors of the combined endpoint at 6, 12 and 24 months, as linearly represented in the survival curves (Log rank).

**Conclusions:**

This risk score enabled the identification of a group with worse prognosis at 12 months.

## Introduction

Heart failure (HF) is a syndrome with high prevalence (1-3% of the population, 5-10% among individuals aged 65-79 years, and 10-20% in older than 80 years), which has been increasing in the last decade due to population ageing and higher survival of subjects suffering from certain diseases, such as ischemic heart disease and arterial hypertension.^1^

HF is characterized by a defective cardiac feeling and/or impairment of blood ejection according to metabolic needs, resulting in a classic constellation of signs and symptoms of pulmonary or systemic congestion.^2,3^

HF is the first cause of early rehospitalizations (in the first 30 days) in elderly individuals. A high rate of readmission for acute HF is observed in the first month after hospital discharge.^4^ Despite the significant increase in hospitalizations due to acute decompensated HF, models of risk stratification in patients hospitalized for acute HF have not been well established.^5^ For this reason, clinical, analytical (including biomarkers) and echocardiographic tools for risk stratification may be useful in the medical decision making.^6^ Among the biomarkers, natriuretic peptides, which are correlated with left ventricular telediastolic pressure (LVTP), usually increased in the HF, are strong prognostic predictors of rehospitalizations and/or death.^7^

LVTP can also be predicted by echocardiography. The assessment of the relationship between mitral ring velocity and transmitral flow velocity curves by tissue Doppler echocardiography provides better estimates of LVTP as compared with other echocardiographic methods^8^.

There are other classical biomarkers with prognostic value in HF. Natremia is inversely correlated with plasma renin activity and is a strong predictor of cardiovascular mortality.^9^ Serum urea and creatinine levels are also predictors of a worse prognostic in HF^10^. Kidney injury in HF generally represents a combination of previous kidney injury, aggravation of renal perfusion, venous congestion and effect of therapy, namely angiotensin-converting-enzyme inhibitor (ACE inhibitor)/ angiotensin II receptor blockers (ARBs), diuretics and mineralocorticoid receptor antagonists (MRAs).^10^

The benefits of the therapy with ACE inhibitors are noticed since the beginning of the therapy that continue in long-term, with greater reduction in the risk of death or rehospitalization for HF in patients with reduced left ventricular ejection fraction (LVEF).^11^

Therefore, despite available data in the literature on long-term prognostic factors in chronic HF, data on (acute or chronic) decompensated HF patients are more scarce.^12^

The aim of this analysis was to develop an AHFR (acute heart failure registry) score, predictor of unfavorable prognostic events in hospitalized patients with acute HF syndromes.

## Methods

### Study design

We designed an observational, retrospective cohort study.

### Study population

The total population consisted of 600 patients hospitalized for acute HF in a cardiology service of a non-tertiary hospital from 2009 to 2011. All patients signed the informed consent form, according to the protocol.

Inclusion criterion was diagnosis of acute HF, defined according to the European Society of Cardiology criteria.^3,13^ Exclusion criteria were high-output HF, high suspicion for acute coronary syndrome as the etiology of HF at hospital admission (including patients requiring urgent reperfusion therapy), acute myocarditis, infectious endocarditis, pulmonary infection, pulmonary arterial hypertension, and severe mitral stenosis. Patients admitted and discharged in the emergency service were also excluded.

### Variables and definitions

Variables of anthropometry, clinical presentations, comorbidities, precipitating factors, echocardiographic measurements, intra-hospital treatment and medications prescribed at discharge were included.

Data collection and electrocardiography were conducted at patient's admission in the emergency service.

Anemia and chronic kidney disease (CKD) were defined according to the National Kidney Foundation as hemoglobin ≤12g/dL for men and postmenopausal women, and estimated glomerular filtration rate (eGFR) calculated by the Modification of Diet in Renal Disease (MDRD) equation lower than 60 mL/min/1.73m^2^ prior to hospital admission.

Hypertensive crisis was defined as a relatively abrupt and symptomatic rise in systolic arterial pressure ≥ 180 mmHg and/or diastolic arterial pressure ≥ 110 mmHg.

Non-hypertensive acute pulmonary edema (APE) was defined as a gradual or sudden onset of dyspnea, tachypnea, hypoxemia and/or radiologic changes compatible to pulmonary edema, and not precipitated by severe hypertension.

Arrhythmia was defined as sustained ventricular tachycardia, atrial fibrillation (AF) or flutter with rapid response or any other supraventricular tachycardia. HF with preserved LVEF, evaluated approximately 72 hours after hospital admission for decompensated HF, was defined as the presence of HF signs and symptoms and LVEF higher than 50% and/or atrial dilation, mitral inflow E/A ratio <1 or >2, E/e' ratio >15.^14^ HF caused by valve heart disease included moderate or severe valve disease. Multifactorial HF refered to multiple anomalies; it is not possible to identify the main one.

### Endpoints

Clinical follow-up of patients were performed up to 24 months (median time [interquartile range]. The primary endpoint for score derivation was defined as all-cause mortality and/or rehospitalization for HF at 12 months. For score validation, the following endpoints were used: (i) all-cause mortality and/or (ii) rehospitalization for HF at 6, 12 and 24 months of clinical follow-up.

### Echocardiographic study

Transthoracic echocardiography was conducted during hospitalization (mean of 3.2 ± 2.8 days of hospital admission) with a GE Vivid 7® echo machine. LVEF was determined by the biplane Simpson's method. The echocardiographic parameters 'estimated pulmonary artery systolic pressure' (PASP) and 'E/e' ratio' were also evaluated in the study.

### Statistical analysis

Continuous variables were reported as mean and standard deviation, and percentage of patients in the intervals obtained with the cutoff points. Categorical variables were described as absolute and relative frequencies (%).

The Student's t-test was used for continuous variables (that had previously passed the Kolmogorov-Smirnov normality test) and the chi-square test for comparisons between categorical variables.

Logistic regression analysis and Cox regression were performed when appropriate (95% confidence interval). A significance level of p<0.05 was adopted.

**Figure 1:**
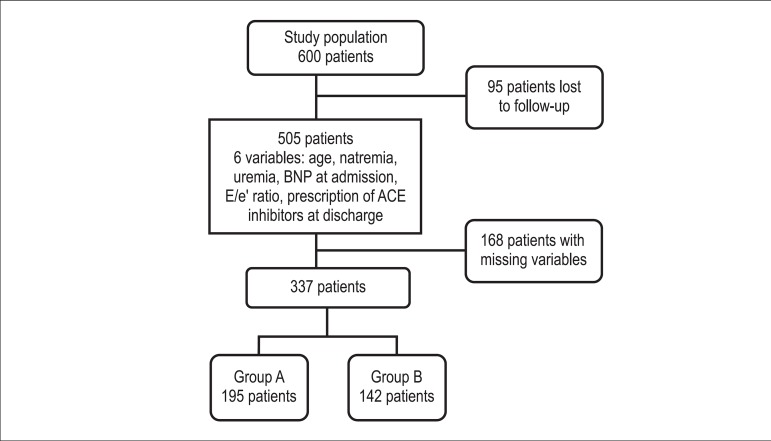
Diagram of study design with number of patients and number of variables.

Of the 600 patients included, 95 were lost to clinical follow-up. In the population of 505 patients, six independent, predicting variables of the event (death/rehospitalization for HF) were identified using the endpoint in 242 patients. Then, 337 patients were classified according to the risk score as Group A (lower risk) or Group B (higher risk) (Figure 1).

It is important to assess this prognostic score regarding its discrimination and calibration. Discrimination was estimated by the area under the curve (AUC), and calibration was estimated by the Hosmer-Lemeshow test.

All analyses were performed by the Statistical Package for The Social Sciences (SPSS) software, version 18.0.

## Results

### Characterization of the study population

Clinical characteristics of the patients from whom the score was obtained are shown in table 1. Clinical, analytical and echocardiographic markers that were independent predictors of the primary endpoint (death for any cause a/or rehospitalization at 12 months of clinical follow-up) were determined by Cox regression analysis. These markers corresponded to the variables included in the score (Table 2).

**Table 1:** Characterization of the study population and predicting variables of mortality and/or rehospitalization at 12 months

Characteristics		Without endpoint at 12 M (n=263)	With endpoint at 12 M (n=242)	p value *
Age >75 years (%)		55.5	70.2	< 0.001
Female (%)		46.4	52.7	0.03
BMI (Kg/m^2^) mean ± SD		28.2±4.9	26.2±5.2	0.01
Risk/etiologic factors and associated comorbidities (%)	Diabetes mellitus	36.1	36.8	NS
	Arterial hypertension	29.7	25.6	NS
	Dyslipidemia	30.8	21.1	NS
	Previous AMI	14.8	14.5	NS
	Previous CTS	9.1	12	NS
	Stroke	7.2	7.4	NS
	Atrial fibrillation	48	53.5	0.01
	CKD	21.0	42.3	0.02
	Anemia	33.8	43.4	0.02
Clinical parameters (%)	SAP < 140 mmHg	52.5	61.2	0.04
	Mean AP < 95 mmHg	41.9	50.6	0.04
	BMI > 30Kg/m2	32.8	24.8	NS
	HR > 100 bpm	27	38.5	0.01
Radiologic parameters (%)	Pulmonary edema	45.1	54.7	0.03
Laboratory parameters (%)	Hyponatremia (< 135 mmoL/mL)	14.4	22.3	< 0.01
	BNP ≥ 400 pg/mL at admission	48.3	61.4	< 0.01
	Urea ≥ 60 mg/dL	39.2	52.5	< 0.01
	eGFR (MDRD) < 60 mL/min/1.73m2	52.5	66.1	0.01
	BNP at discharge ≥ 400 pg/mL	27.3	46.0	< 0.01
Echocardiographic parameters (%)	E/e’ ratio > 15	38	56.6	< 0.01
	LVEF < 35%	23.6	24.8	NS
	PASP > 50 mmHg	26.6	41.7	0.01
Medication at discharge (%)	Loop diuretics	96.6	97.9	NS
	Mineralocorticoid receptor antagonists	42.2	46.9	NS
	ACE inhibitor/ARBs	86.7	76.3	< 0.01
	BB	44.5	37.3	NS
	Statins	40.7	35.9	NS
	BB or ACE inhibitors	69.6	66.5	NS

ARBs: angiotensin II receptor blockers; ACE: angiotensin-converting-enzyme; AMI: acute myocardial infarction; AP: arterial pressure; BB: beta-blockers; BMI: body mass index ; BNP: brain natriuretic peptide; CTS: cardiothoracic surgery; CKD: chronic kidney disease; eGFR: estimated glomerular filtration rate; HR: heart rate; LVEF: left ventricular ejection fraction; MDRD: modification of diet in renal disease; PASP: pulmonary artery systolic pressure; SAP: systolic arterial pressure;

(*) between-group comparison.

**Table 2:** Independent predictors of primary endpoint (mortality and/or rehospitalization for heart failure at 12 months of follow-up) by Cox multivariate regression analysis

Variables	HR	Confidence Interval (95%)	p value	Score
Age ≥ 75 years	1.7	1.1-2.5	0.01	2
E/e' ratio ≥ 15	1.6	1.1-2.3	0.009	2
BNP ≥ 400 pg/mL	1.37	1.0-1.9	0.04	1
Uremia ≥ 60 mg/dL	1.15	1.0-1.5	0.04	1
Natremia < 135 mEq/L	1.37	1.0-1.8	0.03	1
Without ACE inhibitor/ARBs* at discharge	1.9	1.2-2.9	0.004	2

BNP: brain-type natriuretic peptide; ACE: angiotensin-converting-enzyme; ARBs: angiotensin II receptor blockers;

(*) in case of intolerance to ACE inhibitors.

### AHFR score: derivation

**Figure 2:**
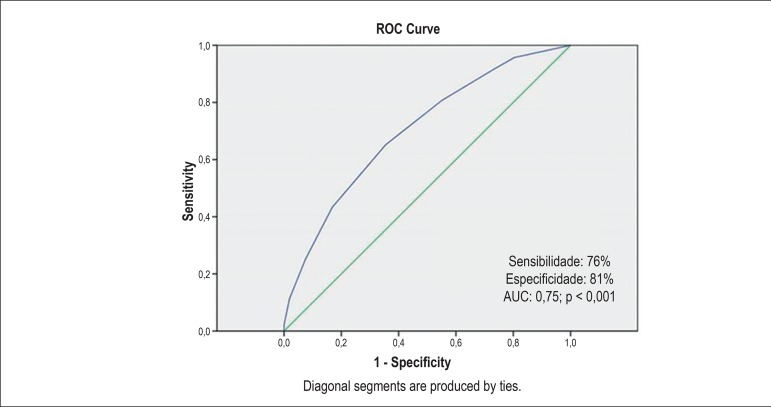
After the total score value was determined, a 4-point cut-off was determined for each ROC curve at 12 months.

One or two points (p.) were given according to the odds ratio (OR) obtained (p<0.05); 1 point for OR<1.5 and 2 points for OR>1.5. The maximum total score was 9 points (Table 2). After calculation of total score, a 4-point cut-off was determined for each ROC curve at 12 months (Figure 2). Two groups were formed based on the number of points, group A (n=195) < 4 points versus group B (n=142) ≥ 4 points.

### AHFR score: validation

Clinical, analytical, echocardiographic parameters as well as event rate (death for any cause and/or rehospitalization for HF) at 6, 12 and 24 months were compared between the two groups.

Therefore, the area under the ROC curve for the endpoint (mortality and/or rehospitalization at 12 months) was 0.74, with an intermediate discrimination score. The score was predictor of the event (p<0.001), with 65% accuracy.

In table 3, comparisons of the two groups according to the AHFR score are found. Group B was composed of older patients, with lower body mass index and higher prevalence of kidney disease. Other risk factors for anemia observed in the study group included female gender and CKD.^15^ In addition, group B had lower eGFR than group A (p<0.001).

**Table 3:** Clinical characterization by risk groups

Characteristics		Group A (n=195)	Group B (n=142)	p value *
Age (mean ± SD)	Mean	75.2±9.6	80.1±9.6	< 0.001
	Women	77.2±8.2	81.6±7.9	0.05
	Men	73.3±10.2	78.5±11.0	0.002
Female (%)		49.2 (n=195)	52.1(n=142)	0.6
Mean BMI (Kg/m^2^) ± SD		28.2±4.9	26.2±5.2	0.01
Mean RICA score ± SD		2.4±1.4	5.8±1.3	<0.001
Risk/etiologic factors and associated comorbidities (%)	DM	38.5	32.4	0.25
	Arterial hypertension	72.8	57.0	0.003
	Dyslipidemia	30.8	21.1	0.048
	Known CHD	36.9	38.7	0.7
	Previous AMI	13.4	17.4	0.6
	Previous CTS	4.6	10.6	0.03
	Stroke	9.7	7	0.38
	AF	50	42.3	0.03
	CKD	21.0	42.3	<0.001
	Anemia	37.4	57.7	<0.001
Clinical presentation of HF (%)	Decompensated HF	67.7	72.5	0.01
	APE (nh)	13.3	11.3	
	APE (h)	16.9	7.7	
	Cardiogenic shock	0.5	2.8	
	Right HF	1.5	5.6	
Precipitating factors (%)	Ischemia/type 2 ACS	11.8	14.1	0.03
	Cardiac arrhythmias	22.1	16.9	
	Hypertensive crisis	15.9	7.0	
	Multifactorial (renal dysfunction, anemia, infection, poor compliance to therapy, diet and others)	50.3	62.0	
HF subtypes (%)	HF with decreased LVEF	47.7	59.6	
	HF with preserved LFEV	52.3	40.4	
	Hypertensive heart diseases (including those associated with AF and DM)	33.3	28.9	0.02
HF etiology (%)	Ischemic CM	25.6	40.1	
	Non-ischemic DCM	21.0	15.5	
	Valve disease	11.8	8.5	
	Cor pulmonale	3.6	6.3	
	Multifactorial	4.6	0.7	
Parameters at admission				
AP (mean ± SD)	SAP (mmHg)	146.2±30.5	130.9±29.5	< 0.001
	DAP (mmHg)	83.7±19.9	75.4±16.0	<0.001
Laboratory				
	eGFR (mL/min) mean± SD	51.0±21.8	37.7±17	< 0.001
	eGFR MDRD < 60 mL/min/1.73m^2^ (%)	21	42.3	<0.001
	Sodium < 135 mmoL/L (%)	7.7	29.6	< 0.01
	Potassium (mmol/L) mean ± SD	4.5±0.6	4.7±0.7	< 0.01
	Hemoglobin (g/dL) mean± SD	13.0±2.0	12.2±2.1	< 0.001
	RDW > 15 (%)	49.7	68.3	0.02
	BNP > 400 pg/mL at admission (%)	31.8	43.7	< 0.001
	BNP > 400 pg/mL at discharge (%)	10.3	37.3	< 0.001
	PCR (mg/dl) mean ± SD	1.8±2.4	2.5±3.4	0.02
Echocardiographic				
	Mean LVEF (%)	50.1±15.1	46.0±16.8	0.02
	LVEF < 30% (%)	10.8	17.0	0.04
	LVEF 30-44% (%)	28.7	35.5	0.04
	LVEF ≥ 50% (%)	52.3	40.4	0.04
	PASP (mmHg) mean± SD	42.2±12.9	50.0±14.7	< 0.001
Medication at discharge (%)				
	BB	46.2	33.1	0.02
	ACE inhibitors	68.7	52.1	< 0.001
	ARBs	24.1	12.7	< 0.001
	BB+ ACE inhibitors/ARBs	44.1	23.9	0.04
	Furosemide	95.4	96.5	0.6
	Spironolactone	39	46.5	0.1

ACE: angiotensin-converting-enzyme; ACS: acute coronary syndrome; AF: atrial fibrillation; AHFR: acute heart failure registry; AMI: acute myocardial infarction; APE (h): acute pulmonary edema (hypertensive); APE (nh): acute pulmonary edema (non-hypertensive); ARBs: angiotensin II receptor blockers; BB: beta-blockers; BMI: body mass index; CAD: coronary artery disease; CG: Cockcroft-Gault; CKD: chronic kidney disease; CM: cardiomyopathy; CTS: cardiothoracic surgery; DAP: diastolic arterial pressure; DCM: dilated cardiomyopathy; DM: diabetes mellitus; eGFR:- estimated glomerular filtration rate; LVEF: left ventricular ejection fraction; HF: heart failure; MDRD: modification of diet in renal disease ; PCR: protein chain reaction; PASP: pulmonary artery systolic pressure; RDW: red cell distribution width; SAP: systolic arterial pressure; SD: standard-deviation;

(*) comparison between the risk groups (A e B).

With respect to electrocardiographic changes, AF rhythm was predominant in group A, whereas other non-sinus rhythm was predominant in group B (p<0.01). No statistically significant differences in intraventricular conduction (QRS) duration were detected between the groups.

The identification of the precipitating factor is crucial for patient's stabilization.^16,17^ In our study, the most frequent precipitating factor was multifactorial (including low compliance to diet and therapy) in both groups.

The most frequent HF etiology was ischemic heart disease (40.1%).

In group B, we found a high proportion of older patients non-adherent to the therapy and posology proposed.

In group B, nearly 40% of patients had LVEF higher than 50%. Other echocardiographic parameters are described in table 3.

Medication started during hospitalization was considered of higher relevance and prognostic impact. At hospital discharge, approximately 52% of patients in group B were receiving ACE inhibitors and 46% spironolactone, and 20% of them had LVEF lower than 30%. Medications received by the patients at discharge are described in table 3.

Mean hospital stay duration was 8.6 ± 7 days, with a mean of 10 (±7.7) days in group B.

AHFR score as predictor of events during clinical follow-up

Rehospitalization rates at 6, 12 and 24 months are shown in table 4.

**Table 4:** Rate of rehospitalization for heart failure at 6, 12 and 24 months by risk groups

Rehospitalization (%)	Group A	Group B	OR (IC 95%)	p value
6 months	21.5	30.5	1.6 (1.2-2.6)	0.04
12 months	34.7	44.4	1.6 (1.2- 2.5)	0.04
24 months	48.2	58.7	1.5 (0.9-2.4)	0.06

**Figure 3:**
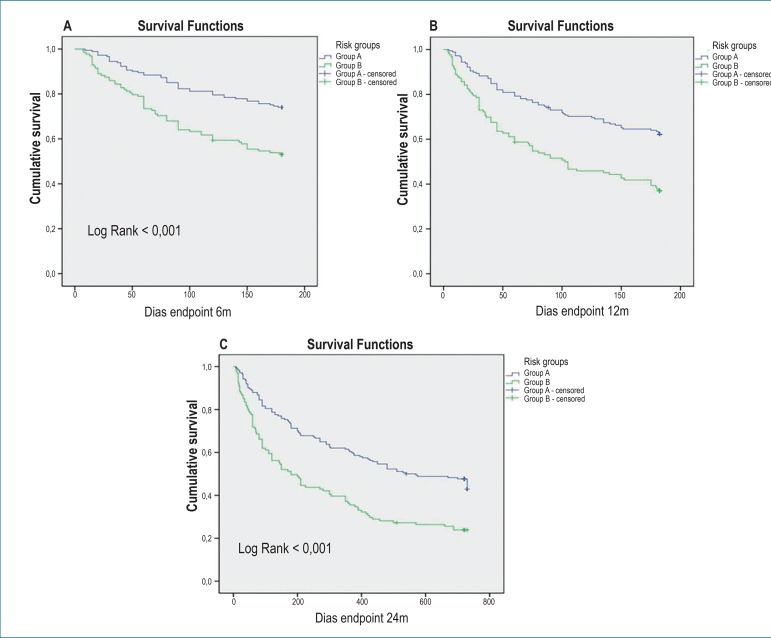
Kaplan Meier curves showing the rate of combined endpoint (mortality and/or rehospitalization) of group A (score < 4) and group B (score > 4) at 6 (A), 12 (B) and 24 months (C) of clinical follow-up.

Nearly one-fourth of patients in group B were rehospitalized within 90 days after hospital discharge. Approximately 25% of patients in group A and 50% in group B reached the endpoint at 6 months after discharge (p<0.001). However, in both groups, the rehospitalization rate for decompensated HF and/or all-cause mortality was higher at three months after discharge, as indicated in the Kaplan Meier curves (Figures 3A, B and C).

## Discussion

In this retrospective study, a new risk score for medium-term events was constructed in patients hospitalized for acute HF syndrome. The inclusion of four variables previously identified in risk models and the identification of two new variables - E/e' ratio and lack of ACE inhibitor/ARBs (for those intolerant to ACE inhibitors) prescription at discharge - enabled the identification of a high-risk group, with score higher than 4 (group B). This group was mostly constituted of older patients, who exhibited higher number of comorbidities, higher hemodynamic instability at admission, higher left ventricular dysfunction and worse prognosis in short, medium and long term.

The identification and clinical characterization of a higher risk group for events facilitates an earlier multidisciplinary approach, and promotes the correct identification of decompensating factors, higher therapy compliance, and reduced hospital stay, hospital morbimortality and readmission that consume most of the resources involved in this syndrome.^5^

Four of the variables included in the score developed in this study are also present in many models. Nevertheless, our study is original in including an echocardiographic variable (E/e' ratio) determined during hospitalization and another variable determined on the day of discharge (lack of ACE inhibitor/ARBs prescription). As in previous models, this score enabled the identification of a higher risk, older group, with higher number of comorbidities, named cardiorenal syndrome.

The risk models used in acute HF have several particularities. First, patients are assessed at admission or at the emergency service, which generally prioritizes the assessment of a very short- term risk (during hospitalization) or a medium-term risk (from 2 to 6 months after discharge), as the identification of high-risk patients contributes to a closer follow-up and more intensive therapy in this period when patients are more vulnerable.^18^ For this reason, the variables of these models may be similar but are slightly different as compared with those of chronic HF scores, in emphasizing easy, rapidly accessible clinical, demographic and analytical factors.^18^

The aim of this study was to evaluate medium-term risk (12 months) by including a variable different from the majority of the models - the lack of ACE inhibitor/ARBs (if intolerant to ACE inhibitor) prescription at discharge. Although "intra-hospital mortality" or "mortality at 90 days" endpoints seem to be more relevant in acute diseases, including acute HF, long-term follow-up cannot be neglected, and other variables such as evidence-based therapy that has a later impact on the prognosis should be included.

Several prognostic models in the context of acute HF are available in the literature. These models can be classified into three groups: five models were conducted with hospitalized patients, one included hospitalized patients included in clinical trials, and two models were conducted in an emergency service. Table 5 describes the summary of the prognostic models in acute HF.^5,6,19-25^ Three markers included in the score created by us - age, natremia, and uremia - are common in most of the models. The prognosis of acute HF progressively worsens with age, for the effect of age *per se* and for its association with higher comorbidity and frailty.^26^ Renal failure is common among patients with acute HF. Some studies have reported that high levels of urea triplicate the risk for intra-hospital mortality and post-discharge mortality.^10^ With respect to hyponatremia, a multivariate analysis showed that a 3 mmoL/L decrease in case of natremia lower than 140mmoL/L increases the intra-hospital mortality by 19.5%.^9^

**Table 5:** Prognostic models in acute heart failure *

Author	Year of publication	Deriving cut-off (n)	Validation cut-off (n)	Variables (n)	Result/AUC
ADHERE^6^ Fonarrow	2005	International Multicentric (33,046)	Multicentric (32,229)	Age. Clinical Laboratory (4)	IHM/ 0.75
AHFI ^19^ Auble	2005 (derivation) 2008 (validation)	National Multicentric (33,533)	Randomized sample (8,384)	Demographic Clinical Laboratory Non-invasive diagnostic tests (21)	IHM/ 0.59
GWTG-HF ^21^Peterson	2010	International Multicentric Community (27,850)	Multicentric Community (11,933)	Demographic Clinical Laboratory Comorbidities 7)	IHM / 0.75
EFFECT^22^ Lee	2003	National Multicentric (2,624)	Multicentric Community (1,407)	Demographic Clinical Laboratory Comorbidities (10)	Mortality in 30 days /0.79 Mortality at one year /0.76
OPTIMIZE-HF^20^ O’Connor	2008	International Multicentric Registry (4,402)	OPTIME CHF (949) y ESCAPE (433)	Demographic Clinical Laboratory Comorbidities (13)	Mortality in 60-90 days/0.72
OPTIMIZE-HF^5^ Abraham	2008	International Multicentric Registry (37,548)	Internal Bootstrapping ADHERE trial (181,830)	Demographic Clinical Laboratory Systolic dysfunction (7)	IHM/ 0.74
OPTIME CHF^23^ Felker	2004	International Multicentric (949	Internal Bootstrapping	Demographic Clinical Laboratory (5)	Mortality in 60 days/0.77
Otawa^24^ Stiell	2013	National Multicentric Community (507)	Internal Bootstrapping	Clinical laboratory (10)	Mortality in 30 days or non-fatal event in 14 days /BNP 0.77. no BNP 0.75
EHRMG^25^ Lee	2012	National Multicentric Community (7,433)	Multicentric Community (5,158)	Clinical Laboratory Comorbidities (10)	Mortality in 7 days/0.8

AUC: area under the curve; ADHERE: Acute Decompensated Heart Failure National Registry; AHFI: Acute Heart Failure Index; EFFECT: Enhanced Feedback for Effective Cardiac Treatment; EHMRG: Emergency Heart Failure Mortality Risk; GWTG-HF: Get With the Guidelines-Heart Failure; OPTIMIZE-HF: Organized Program to initiate Lifesaving Treatment in Hospitalized Patients with Heart Failure; OPTIME-CHF: Outcomes of a Prospective Trial of Intravenous Milrinone for Exacerbations of Chronic Heart Failure; Ottawa: Ottawa Heart Failure Risk Model;

(*) Adapted from Ferrero P. et al. Int J Cardiol. 2015;188:1-920^18^.

Despite higher availability and proved prognostic utility of natriuretic peptides, these compounds have not been included in most of the risk prediction models. Some studies suggest that an increment by 30% in normal NT-proBNP levels at admission increases by six times the risk of rehospitalization.^27^

The assessment of the relationship of mitral ring velocity with transmitral flow velocity curves (E/e' ratio) by tissue Doppler was found to be an independent predictor of yearly mortality in patients hospitalized for acute HF.^28^

Although the benefits of an early start of ACE inhibitors in acute HF have not been demonstrated in the literature, their prescription is mandatory within the first 48h-72h after admission, with proven benefits in reducing mortality and rehospitaization rate, according to the European Society of Cardiology recommendations.^3^

Despite numerous studies showing that the lack of the prescription of beta-blockers at discharge is a mortality predicting factor, this was not observed in this study, probably due to a selection bias.

The estimated risk at hospital admission may help to decide whether or not a patient is candidate for intensive therapy. However, several studies have shown that risk scores estimated on the day of discharge (including biomarkers and therapy prescribed at discharge) have better prognostic value as compared with those determined at admission.^29^ Risk predicting tools are crucial to determine the prognosis in HF. Although these risk models can precisely determine short-term prognosis of acute HF, they should be extensively tested in elderly patients or those with multiple comorbidities.^29^ Besides, prospective, randomized studies are needed to establish the impact of long term risk stratification on acute HF patients.^29^

### Limitations

Some limitations inherent in the construction of this score should be considered in the interpretation of the results. The fact that this is a retrospective study opens up the possibility of selection bias. An external validation of the model is needed, preferentially in another center. The diagnosis of acute HF was based only on the European Society of Cardiology criteria and the date of onset of symptoms was not determined. For this reason, it is not possible to differentiate *de novo* acute HF from acutely worsened chronic HF. In addition, analysis of treatment and prognosis should be adjusted because of the heterogeneity of the sample. Another limitation refers to the fact that we did not include patients discharged home from the emergency department. Also, there was a large number of missing variables when the completion of data was optional, which affected the results. The echocardiography was performed some days post-admission, rather than on the day of admission, which may influence the measurements used in the score construction.

## Conclusions

In this study, we constructed a new risk score of medium-term events in patients hospitalized for acute HF syndrome. The inclusion of four variables previously identified in risk models, in addition to the identification of two additional variables: E/e' ratio and lack of ACE inhibitor/ARBs prescription on the day of discharge enabled the identification of group at high risk for all-cause mortality at 12 months after discharge. This group (group B), with score higher than 4, was mostly constituted of older patients, who exhibited higher number of comorbidities, higher hemodynamic instability at admission, higher left ventricular dysfunction and worse prognosis in short, medium and long term. This group may benefit from a closer monitoring and early start of evidence-based therapy.
